# P-1582. Knowledge of High Consequence Infections Among Veterans Affairs Employees During a Drive Thru Flu-Pod Drive

**DOI:** 10.1093/ofid/ofaf695.1761

**Published:** 2026-01-11

**Authors:** Lisa Bailey, Monique Thorne, Debra Noland, Florence M Ford, Sabrina Mohsin, George Psevdos

**Affiliations:** Northport VAMC, Northport, New York; Northport VAMC, Northport, New York; Northport VAMC, Northport, New York; Northport VA, Northport, New York; Stony Brook University Hospital, Branchburg, NJ; Northport VA Medical Center, Northport, New York

## Abstract

**Background:**

The world has faced several high consequence infectious diseases or infections (HCI) over the past years with outbreaks traveling from country to country. Recent outbreaks such as Marburg virus in Tanzania (2023) Ebola virus in Uganda (2022), Middle East Respiratory Syndrome Coronavirus (MERS) in Saudi Arabia (2018) serve as a frightening reminder that HCI can circulate at any given time, generating considerable public health and economic consequences. Our Veterans Affairs Medical Center (VAMC) has a response HCI plan. We evaluated the knowledge of the existence of this plan and of HCI among health care employees (HCE) receiving the influenza vaccine during a drive-thru flu point of distribution (POD) drive

**Figure 1:**
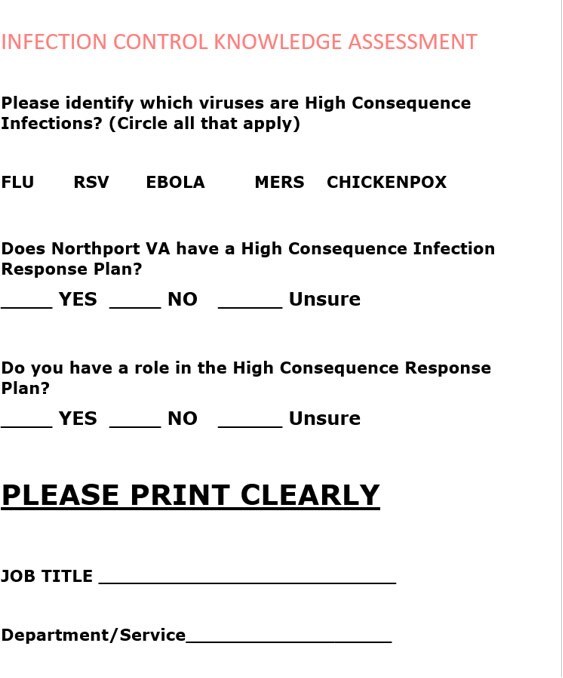
Questionnaire given to employees receiving the influenza vaccine

**Figure 2:**
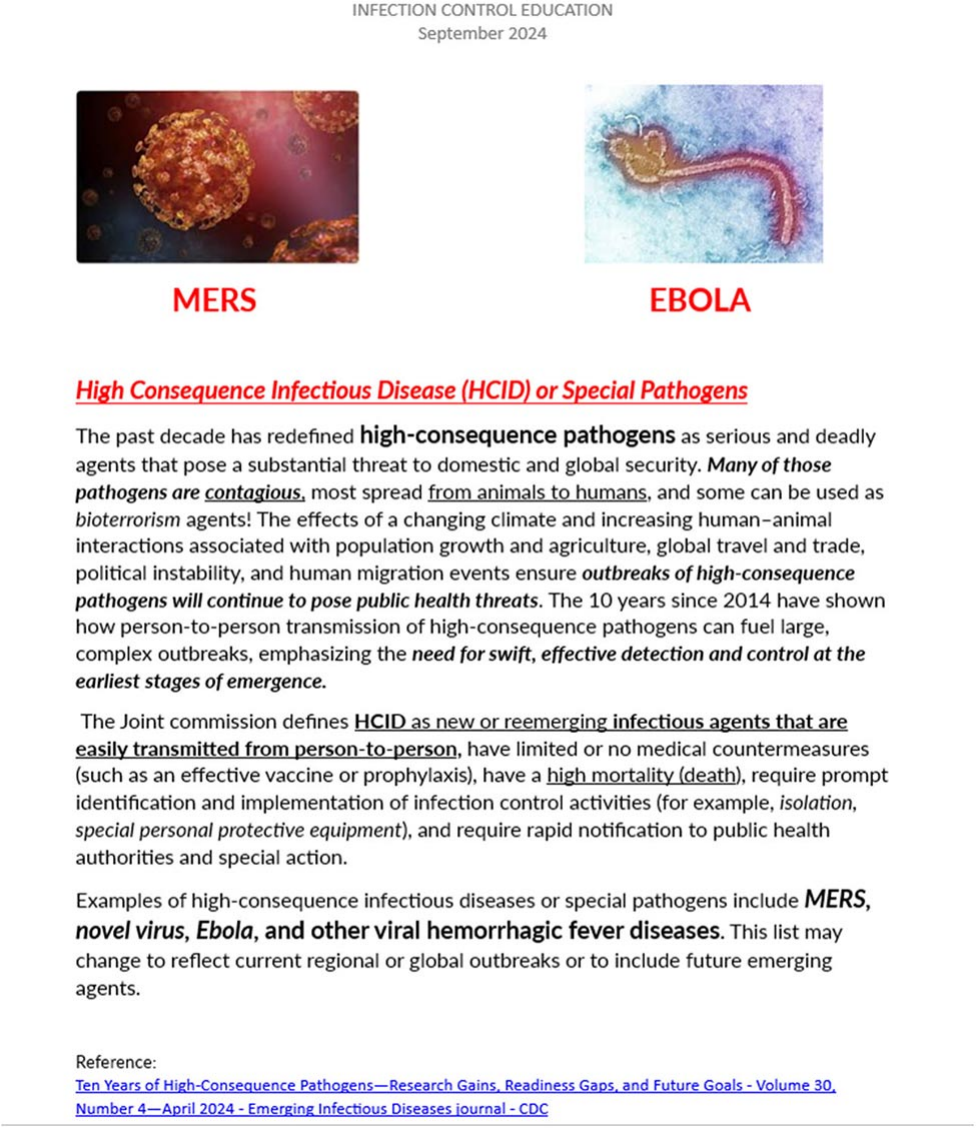
Education Pamphlet given to employees upon receipt of vaccine and completion of questionnaire

**Methods:**

HCE influenza vaccination was planned in the fall of 2024 as a drive-thru flu POD, a single day, 90 min event. The location was the main employee entrance at Northport VAMC. A questionnaire was given to each employee who received the influenza vaccine. Completion was voluntary. The questionnaire is depicted in figure 1. The participants were asked to circle which of the following are HCI: the choices were “FLU, RSV, MERS, EBOLA, CHICHEN POX.” The second question: “Does Northport VA have a HCI response plan, Yes/No/Unsure”. The 3^rd^ question “Do you have a role in the HCI plan, Yes/No/Unsure.” An education pamphlet on MERS and EBOLA was provided at completion of the questionnaire. see figure 2

**Table 1:**
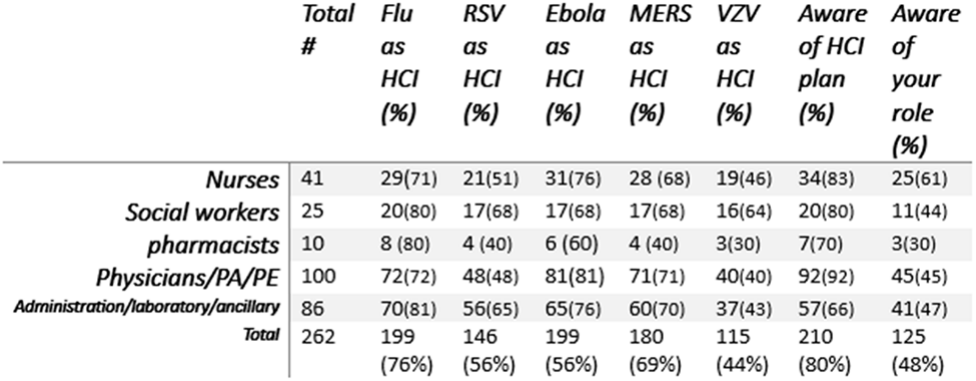
Answers of the Questionnaire

**Results:**

311 employees received the vaccine and 262 completed the questionnaire. Table 1 shows the answers. Equal number of HCE (199) identified the seasonal influenza and Ebola (199) as HCI. 71% of nurses and 72% of physicians/physician assistants/extenders regarded influenza as HCI. While 210 responded of awareness of the facility HCI plan, only 125 were clear of their role in this plan. Fewer identified chicken pox as HCI (115) followed by RSV responses (146). MERS received less votes (180) than influenza

**Conclusion:**

In our project we identified that our fellow HCE exhibited significant knowledge gaps in identifying what constitutes a HCI and what should be their role in event of HCI event in our facility. This is an opportunity for enhanced training and education to be conducted by infection control and emergency management teams as a HCI event occurring in the United States soon may become unavoidable

**Disclosures:**

All Authors: No reported disclosures

